# Neglected SARS-CoV-2 variants and potential concerns for molecular diagnostics: a framework for nucleic acid amplification test target site quality assurance

**DOI:** 10.1128/spectrum.00761-23

**Published:** 2023-10-10

**Authors:** Gregory R. McCracken, Daniel Gaston, Janice Pettipas, Allana Loder, Anna Majer, Elsie Grudeski, Geneviève Labbé, Bryn K. Joy, Glenn Patriquin, Jason J. LeBlanc

**Affiliations:** 1 Division of Microbiology, Department of Pathology and Laboratory Medicine, Nova Scotia Health (NSH), Halifax, Nova Scotia, Canada; 2 Department of Pathology, Faculty of Medicine, Dalhousie University, Halifax, Nova Scotia, Canada; 3 Division of Hematopathology, Department of Pathology and Laboratory Medicine, Nova Scotia Health (NSH), Halifax, Nova Scotia, Canada; 4 Nova Scotia Provincial Public Health Laboratory Network (PPHLN), Halifax, Nova Scotia, Canada; 5 National Microbiology Laboratory (NML), Public Health Agency of Canada (PHAC), Winnipeg, Manitoba, Canada; 6 Medical Sciences Program, Faculty of Sciences, Dalhousie University, Halifax, Nova Scotia, Canada; 7 Department of Medicine (Infectious Diseases), Faculty of Medicine, Dalhousie University, Halifax, Nova Scotia, Canada; Quest Diagnostics, San Juan Capistrano, California, USA

**Keywords:** quality, COVID-19, SARS-CoV-2, PCR, mutation, NAAT, sequence, target, variant, genome

## Abstract

During the COVID-19 pandemic, SARS-CoV-2 detection using nucleic acid amplification tests (NAATs) played a key role in clinical management and public health interventions. However, mutations could jeopardize NAAT-based detection if they occur in the NAAT target site, potentially resulting in false negative results. However, mutation monitoring is challenged as the exact location of commercial NAAT target sites is not divulged by manufacturers. This study sequenced commercial SARS-CoV-2 NAAT target sites to assess the impact of mutations occurring in these regions. The resulting sequences for the Xpert, Cobas, and ID NOW SARS-CoV-2 assays were queried against SARS-CoV-2 genome databases to identify mutations in circulating strains. Synthetic DNAs and clinical specimens harboring NAAT target site mutations were used to assess mutation impact. Of 17,600 NAAT target site mutation occurrences in a genome database, 269 compromised target detection. These represented 24 unique mutations that reduced NAAT target sensitivity and nine led to target detection failure. Only seven of these mutations were previously recognized. Overall, this reactive strategy along with passive surveillance identified 29 novel mutations that compromised detection with Xpert and Cobas targets. Knowledge of commercial NAAT target sites, paired with a strategy for mutation impact assessment and ongoing genetic surveillance, provided a robust framework for commercial NAAT target site quality assurance. The question remains of who should be responsible for NAAT target site quality assurance, but collaborative efforts between methods users, industry, and regulatory agencies would be ideal.

**IMPORTANCE:**

Molecular tests like polymerase chain reaction were widely used during the COVID-19 pandemic but as the pandemic evolved, so did SARS-CoV-2. This virus acquired mutations, prompting concerns that mutations could compromise molecular test results and be falsely negative. While some manufacturers may have in-house programs for monitoring mutations that could impact their assay performance, it is important to promptly report mutations in circulating viral strains that could adversely impact a diagnostic test result. However, commercial test target sites are proprietary, making independent monitoring difficult. In this study, SARS-CoV-2 test target sites were sequenced to monitor and assess mutations impact, and 29 novel mutations impacting SARS-CoV-2 detection were identified. This framework for molecular test target site quality assurance could be adapted to any molecular test, ensuring accurate diagnostic test results and disease diagnoses.

## INTRODUCTION

Access to next-generation sequencing (NGS) technologies during the COVID-19 pandemic allowed worldwide monitoring of SARS-CoV-2 variants with mutations conferring increased transmissibility, altered disease epidemiology, severity, or presentation, or decreased effectiveness in control measures such as vaccines, therapeutics, or diagnostics (1 – 3). Depending on the strength of evidence supporting these properties, emerging SARS-CoV-2 lineages have been classified by the World Health Organization (WHO) as variants of concern (VOCs), variants of interest (VOIs), or variants under monitoring (VUMs) (2). To date, no SARS-CoV-2 variants have been assigned based on decreased effectiveness or failures in diagnostics. Despite playing a key role in patient management and public health and infection control mitigation strategies (1), SARS-CoV-2 mutations that could potentially compromise nucleic acid amplification tests (NAATs) were often only reported as incidental findings during diagnostic testing or method comparisons (4).

Accreditation bodies and organizations like the American Society for Microbiology recommend routine quality control and quality assurance practices in clinical and public health laboratories to ensure accurate test results. However, ongoing verification of the appropriateness of NAAT target sites is often overlooked. NAAT target sites are generally designed in genomic regions that are highly conserved, yet SARS-CoV-2 accumulated mutations at a rate between 10^−3^ and 10^−4^ nucleotide substitutions/site/year, raising concerns for potential false negative results (5 – 7). Common NAAT targets for SARS-CoV-2 detection included: spike (S), envelope (E), nucleocapsids (N), open reading frame 1ab (Orf1ab), and more precisely, the RNA-dependent RNA polymerase (RdRp) located within Orf1ab (1). Depending on the number and position of nucleotide mismatches between NAAT target sites and the genome sequences of circulating SARS-CoV-2, these mutations could result in no impact, reduced sensitivity, or target detection failure (4). For example, a 69–70 amino acid deletion (nucleotide positions 21,765 to 21,770) in the alpha and omicron VOCs was shown to cause S gene target failure with the ThermoFisher TaqPath COVID-19 Combo Kit (8); a C26340T mutation was associated to E gene target failure with the Roche Cobas SARS-CoV-2 test (9), and various mutations have been associated to N2 gene target failure on the Cepheid Xpert Xpress SARS-CoV-2 test including C29179T (10 – 12), C29200A (13), C29200T (14 – 16), and C29203T (17). Other NAAT target site mutations reduce target detection sensitivity such as the G15006T (18) and T15009C (19) compromising the Roche Cobas assay targets Orf1ab, G26372T (20) impacting the Cobas E gene target, or the G29195T (21) and G29197T (22) mutations impacting the Xpert N2 gene target.

**Fig 1:**
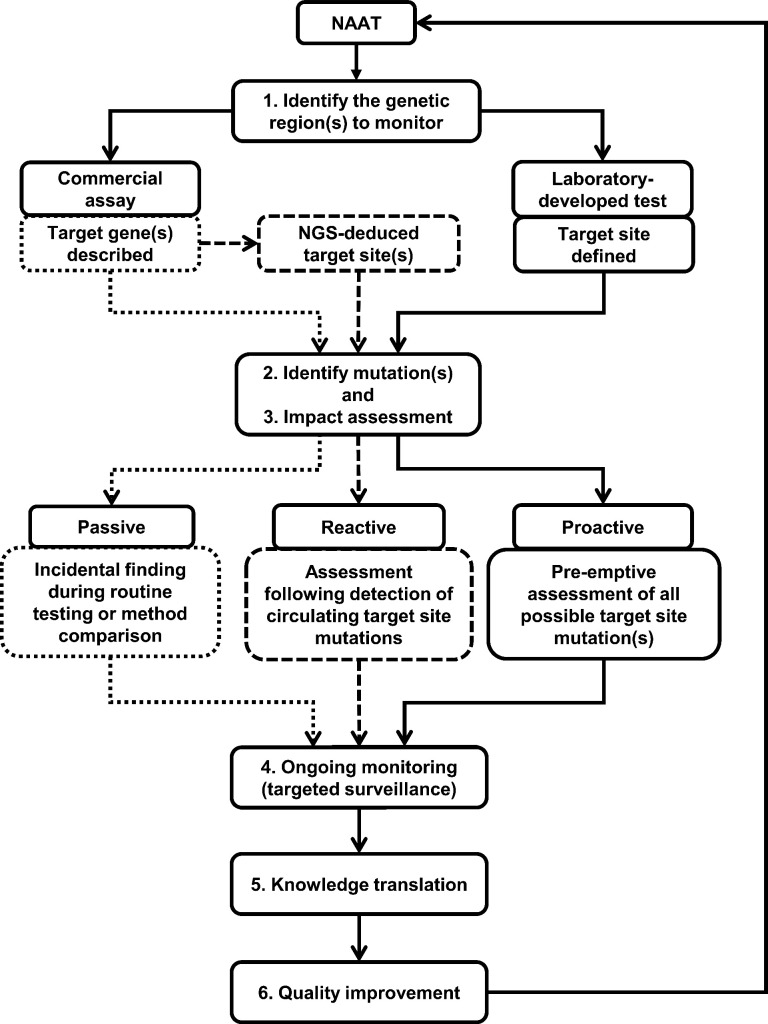
Framework for commercial NAAT target site quality assurance. Solid lines are ideal pathways, dotted lines are possible but inefficient pathways, and dashed lines are practical solutions. Abbreviations: NGS, next-generation sequencing; NAAT, nucleic acid amplification tests.

Mutations impacting diagnostic NAATs are not unique to SARS-CoV-2 and have been reported for parasites (23), fungi (24), bacteria (2,25), and other viruses (26 – 29). With the potential to cause false negative results with molecular technologies (4), the need for NAAT target sites quality assurance is well justified. Ongoing monitoring for NAAT target site mutations would require a quality assurance program that includes: (i) knowledge of NAAT target site(s); (ii) an infrastructure supporting genetic surveillance; (iii) a mutation impact assessment strategy; (iv) ongoing targeted surveillance to understand local epidemiology; (v) a communication strategy to various stakeholders; and if needed, (vi) NAAT reformulation through quality improvement initiatives (Fig. 1).

SARS-CoV-2 is an ideal model to demonstrate a framework for NAAT target site quality assurance, with over 15.5 million genome sequences submitted the EpiCoV database of the Global Initiative on Sharing All Influenza Data (GISAID) (https://gisaid.org/) (30). These genomes can be interrogated with NAAT target site sequences to reveal sequence mismatches. For laboratory-developed tests (LDTs), primer or probe sequences defining the target site(s) are known, which facilitates this task (31 – 33). In contrast, commercial NAATs manufacturers do not divulge the exact location of their target sites and only the gene(s) name is provided. Monitoring mutations occurring in entire genes is possible but adds significantly to the workload and expense, particularly for large targets like SARS-CoV-2 Orf1ab that exceeds two-thirds of the SARS-CoV-2 genome (1).

This study aimed to: (i) identify the target site locations of three commonly used commercial NAATs (34, 35); (ii) query these regions against SARS-CoV-2 genome databases to reveal any mutations; (iii) evaluate mutation impact; (iv) and communicate findings of any mutations compromising target detection. This proof-of-principle framework for commercial NAAT target site quality assurance could be a useful model for other NAATs, targets, and applications.

## RESULTS

### Identification of NAAT target sites

Using NGS on amplicons derived from the Cepheid Xpert (34), Roche Cobas 6800 (34), or Abbott ID NOW (35) SARS-CoV-2 assays, target sites were identified in high-quality data with sequence depth reads ranging from 6,665 to 99,381. Compared to manufacturer-described gene targets, the sizes of the NGS-defined target sites were 1.5- to 151-fold smaller, thereby reducing the genomic regions to monitor for mutations during genetic surveillance (Table 1).

**TABLE 1:** Comparison of manufacturer-described target genes and target sites estimated by amplicon sequencing

NAAT	Manufacturer target gene(s)		NGS-estimated target site(s)		Fold reduction in size^*c*^
	Name (genome location^*a*^)	Size (bp)^*a*^	Name (genome location^*a*^)	Size (bp)^*a*^	
Cobas (Roche)	Orf1ab (266–21,255)	21,290	RdRp^*b*^	141	151.0
	E (26,245–26,472)	228	E^*b*^	155	1.5
Xpert (Cepheid)	N (28,274–29,533)	1,260	N (29,164–29,230)	67	18.8
	E (26,245–24,472)	228	E (26,269–26,381)	113	2.0
ID NOW (Abbott)	RdRp (13,442–16,237)	2,796	RdRp^*b*^	36	77.7

^*a*^ Genome location and size were determined by pairwise Basic Local Alignment Search Tool alignment against the Wuhan-Hu-1 reference genome (NC_045512.2).

^*b*^ The exact location of the Cobas and ID NOW SARS-CoV-2 target sites was sequenced for mutation monitoring and impact assessments but are not presented. For the Xpert assay, the sequences of the E and N2 genes are identical to those previously presented by Miller et al. (16).

^*c*^ Fold reduction in size represents a ratio between the NGS-estimated target site, and the size of the gene described by the manufacturer. Abbreviations: bp, base pair; E, envelope; NAAT, nucleic acid amplification tests; N, nucleocapsid; NGS, next-generation sequencing; Orf, open reading frame; RdRp, RNA-dependent RNA polymerase.

### Passive genetic surveillance

In Nova Scotia, a subset of weekly positive SARS-CoV-2 specimens is subjected weekly for genome sequencing for surveillance purposes. In some cases, specimens were targeted for sequencing, such as those where a NAAT target detection failure had occurred or where reduced sensitivity was suspected (from a difference in threshold cycle [Ct] values greater than expected between two NAAT targets). Using passive genetic surveillance, four specimens revealed previously recognized mutations that compromised detection for the Cobas E gene (i.e., C26340T) or Xpert N2 gene target (i.e., G29179T, C29197T, and C29200T; Table S1). In addition, two other mutations T15009C and C26388T were observed that reduce the sensitivity of detection for the Roche Cobas Orf1ab (RdRp) and E gene targets, respectively (Table S1). A single specimen with the RdRp T15009C mutation was from the SARS-CoV-2 omicron VOC lineage BA.2.12.1 and resulted in a Ct shift of 4.3 greater than expected between the Cobas assay Orf1ab and RdRp targets (Table S1). The C26388T E gene mutation was previously unrecognized, but following public health investigations, 12 epidemiologically linked cases were identified from the SARS-CoV-2 alpha VOC lineage B.1.1.7, all showing Ct differences between E and RdRp ranging from 6.0 to 10.6 (Table S1).

### Reactive genetic surveillance

In the reactive genetic surveillance approach, NAAT target site sequences were queried for mismatches against SARS-CoV-2 genomes in GISAID, and mutation impact assessments were evaluated using synthetic DNA harboring the mutation of interest. Of 114 total occurrences for 23 unique mutations, none impacted RdRp detection with the ID NOW (Table 2; Table S2). For the Cobas assay, 733 and 8088 mutation occurrences were observed in the RdRp and E gene targets, which consisted of 74 and 73 unique mutations, respectively (Table 2). Of these, only C26340T impacted Cobas E gene detection, but eight occurrences of this mutation were observed in GISAID at the time of analysis (Table 2). For the Xpert E gene target, 7,998 mutation occurrences were observed but none compromised target detection (Table 2). For the Xpert N2 gene target, 667 mutation occurrences were observed, of which 261 (39.1%) compromised target detection (Table 2). More precisely, 58 unique mutations were common among the reactive and proactive approaches, with 32 (55.1%) compromising N2 gene target detection. Of these, 24 reduced sensitivity, and 8 resulted in target detection failures (Table 3).

**TABLE 2:** Summary of circulating SARS-CoV-2 strains with mutations impacting commercial NAAT target detection^*b*^

NAAT	Target site	Proportion of unique mutations impacting target detection^*a*^	Total occurrences of mutations impacting target detection^*a*^
Cobas (Roche)	RdRp	0.0% (0/74)	0.0% (0/733)
	E	1.4% (1/73)	0.1% (8/8,088)
Xpert (Cepheid)	N2	55.2% (32/58)	39.1% (261/667)
	E	0.0% (0/51)	0.0% (0/7,998)
ID NOW (Abbott)	RdRp	0.0% (0/23)	0.0% (0/114)

^*a*^ Numbers of circulating target site mutations were observed in GISAID on 9 August 2022, using the PrimerChecker software.

^*b*^ Abbreviations: GISAID, Global Initiative on Sharing All Influenza Data; NAAT, nucleic acid amplification tests.

**TABLE 3:** Xpert N2 mutations identified with the reactive and proactive mutation impact assessments^*c*^

Mutation impact		Xpert N2 mutations^*a*^	
		Reactive and proactive approach	Proactive approach only
Target detection failure		C29196G, C29197A, C29197T ^*b*^, C29199A, C29200A ^*b* ^, C29200T ^*b* ^, C29203T ^*b*^, and C29205T	C29196A, C29196T, C29197G, C29198G, C29198A, C29198T, C29199T, C29200G, C29203G, C29203A, G29204T, C29205G, C29205A, T29207G, and T29213G
Reduced sensitivity (fold-reduction)	10^3^–10^4^	None detected	T29213A (3827.9) and T29214G (2082.8)
	10^2^–<10^3^	C29171T (50.5), G29179T (149.0)^*b*^; A29181T (384.1), A29182G (384.1), and A29201G (273.9)	C29199G (195.3), T29213C (808.1), T29214A (293.3), and C29215A (239.3)
	10^1^–<10^2^	A29172G (11.4), G29195T (14.0)^*b*^, C29215T (41.2), T29223C (13.1), G29224C (47.2), G29224T (17.1), and G29227A (12.2)	A29201T (27.5), C29208T (44.1), T29214C (70.8), T29216C (16.0), G29219A (14.0), G29219T (10.0), G29220C (17.1), G29224A (21.0), and G29227C (15.0)
	2–<10^1^	C29177T (2.4), G29179A (5 ), A29182T (2.3), G29202C (3.0), G29202A (5.4), G29204C (5.8), G29204A (3.2), T29206C (2.8), T29206A (5.1), C29218T (3.9), G29227T (8.7), and C29228A (2.1)	G29195A (3.9), A29201C (2.8), T29206G (4.1), T29207A (9.3), C29208G (7.6), C29208A (6.6), A29209G (2.3), C29215G (7.6), T29216G (7.6), T29216A (8.1), T29217A (4.7), T29217C (8.7), T29217G (2.0), C29218G (8.1), C29218A (6.2), G29219C (4.7), G29220A (5.8), G29220T (8.7), A29221C (8.7), A29221T (2.1), A29222T (2.4), A29222C (5.4), T29223A (3.2), T29225C (2.4), C29226A (3.2), and C29226G (5.1)
No change		T29164C, T29164A, T29165C, C29167T, A29169G, C29171A, A29171G, T29173C, T29173G, T29185C, T29185A, A29188T, T29192G, T29192C, T29194C, G29202T, A29209T, G29210T, C29211G, G29212T, G29212A, A29221G, A29222G, C29228T, G29229A, and C29230T	T29164G, T29165G, T29165A, A29166G, A29166T, A29166C, C29167G, C29167A, A29168G, A29168T, A29168C, A29169T, A29169C, A29170G, A29170T, A29170C, A29172T, A29172C, T29173A, T29174G, T29174A, T29174C, G29175A, G29175T, G29175C, G29176A, G29176T, G29176C, C29177G, C29177A, C29178G, C29178A, C29178T, G29179C, C29180G, C29180A, C29180T, A29181G, A29181C, A29182C, A29183G, A29183T, A29183C, T29184G, T29184A, T29184C, T29185G, G29186A, G29186T, G29186C, C29187G, C29187A, C29187T, A29188G, A29188C, C29189G, C29189A, C29189T, A29190G, A29190T, A29190C, A29191G, A29191T, A29191C, T29192A, T29193G, T29193A, T29193C, T29194G, T29194A, G29195C, T29207C, A29209C, G29210A, G29210C, C29211A, C29211T, G29212C, T29223G, T29225G, T29225A, C29226T, C29228G, G29229T, G29229C, C29230G, and C29230A

^*a*^ N2 target sequences were queried against the Global Initiative on Sharing All Influenza Data (GISAID) EpiCoV database (9 August 2022).

^*b*^ Previously recognized mutations impacting N2 target detection.

^*c*^ Note: some mutations were confirmed using archived clinical specimens (underscored). The lineages and results from these investigations are provided in Table S2.

### Proactive genetic surveillance

Next, the Xpert N2 gene target was used to highlight the proof-of-principle for proactive genetic surveillance, given this target has been widely used in LDTs in many countries (31 – 33), including the real-time RT-PCR developed by the Centers for Disease Control and Prevention (15). Synthetic DNA representing 201 possible single mutations for the Xpert N2 gene target site was tested, and 88 (43.8%) compromised target detection: 26.1% (23/88) were N2 gene target detection failures, and 73.9% (65/88) led to a reduction in assay sensitivity (Table 3). Of the 143 mutations unique to the proactive approach, 56 (39.2%) compromised N2 gene target detection, with 15 leading to target detection failure and 41 resulting in reduced sensitivity (Table 3).

### Confirmation of mutation impact deduced from synthetic DNAs

The C26340T mutation associated with Cobas E gene target failure was previously published and observed during reactive surveillance, thereby confirming the only result of synthetic DNAs impacting the Cobas E gene target. Of 58 Xpert N2 gene target site mutations observed by reactive genetic surveillance (Table 3), 23 (39.7%) were also observed in one or more of 40 clinical specimens accessible in local or national biorepositories (Table S3). When tested, 100% (40/40) clinical specimens demonstrated similar (*R* ^2^ = 0.9936) fold reductions in sensitivity as synthetic DNA, thus confirming the findings of 23 different target site mutations (Table S3).

## DISCUSSION

COVID-19 was the first pandemic where NGS technologies were readily available to monitor for SARS-CoV-2 variants. Despite the decreased effectiveness of diagnostic testing being a WHO criterion defining a SARS-CoV-2 VOC, VOI, or VUM (2), ongoing monitoring for mutations that could compromise commercial NAATs remains challenged by the lack of disclosure by manufacturer of the exact location of their target sites. This study revealed target sites for three commercial NAATs and demonstrated strategies for mutation monitoring and impact assessments. This framework for NAAT target site quality assurance identified 26 previously unrecognized mutations that compromised Xpert N2 gene target detection in circulating SARS-CoV-2 strains, confirmed two mutations suspected of compromising the Cobas Orf1ab target (now identified as RdRp), and identified a novel mutation reducing the sensitivity of the Cobas E gene target.

Following amplicon sequencing of commercial NAAT target sites, the regions to monitor for mutations were significantly shortened compared to the manufacturer-described target genes (Table 1). This was particularly evident for the Cobas Orf1ab gene at 21,290 base pair (bp) that was shortened 151-fold to a small 141 bp segment within RdRp, as well as the 36 bp ID NOW target that was shortened 78-fold from the manufacturer described RdRp 2,796 bp target. Miller et al. (15) previously used a similar NGS-based approach to characterize the Xpert E and N2 gene targets, which were the same identified in this study. With access to sequencing technologies, it raises the question of why the precise location of NAAT target sites is not divulged by manufacturers to facilitate mutation monitoring. For example, the T15009C mutation identified by passive surveillance in Nova Scotia was one of two mutations suspected by Rodino et al. (19) to reduce the sensitivity of the Cobas Orf1ab target. Similarly, of the five mutations suspected to cause reduced sensitivity of the Cobas Orf1ab target by Popping et al. (18), knowledge of the RdRp target site reveals G15006T as the culprit. Knowledge of the NAAT target sites and passive surveillance also identified C26388T as causing reduced sensitivity for the Cobas E gene. While passive surveillance identified these and other recognized mutations impacting SARS-CoV-2 target detection (Table 1), strains harboring target site mutations would only be detected if tested using a multi-target assay in which the mutation impacts one of its targets or by comparisons between different NAATs, which would be uncommon during routine diagnostic testing.

The reactive genetic surveillance approach in this study leveraged millions of SARS-CoV-2 genomes in GISAID to reveal NAAT target site mutations, regardless of the method used to identify SARS-CoV-2. Using this strategy, all previously recognized mutations as well as 26 additional mutations that compromise the Xpert N2 gene were identified, including three novel mutations that caused target detection failure (i.e., C29196G, C29199A, and C29205T). In this study, the N gene target had the highest proportion of mutations that compromised target detection at 55.2% (Table 2). The Orf1ab, N, and S genes are known to have the highest mutation rates across the SARS-CoV-2 genome, and the N gene has the highest mutation frequency at 202 variations per kilobase (5 – 7). The reason for such a high proportion of N gene mutations might be attributed to it having the highest GC content in the SARS-CoV-2 genome at 48% (compared to approximately 38% across the genome) (5 – 7). With the substitution of G or C for A or T, there would be a reduction in hydrogen bonds and possibly destabilizing primer and probe hybridization to their target sequence (31 – 33). Overall, the reactive surveillance has merits in leveraging genomic surveillance to help identify mutations that could potentially impact diagnostic testing; however, public genome databases rely on voluntary submissions and likely only represent a small subset of circulating viruses. As such, the true burden of NAAT target site mutations may be underestimated.

Proactive surveillance is used to help characterize the impact of NAAT target site mutations that have yet to be observed in circulating SARS-CoV-2 strains. This study also demonstrated a proof-of-concept for proactive genetic surveillance using all 201 possible single-target mutations of the Xpert N2 gene target as an example, prior to database queries to assess their presence in SARS-CoV-2 genomes. All mutations identified by the reactive approach were predicted with the proactive approach, and an additional 83 deleterious mutations remain to be confirmed. Being able to predict mutation impact prior to circulation would be particularly appealing for rapidly evolving targets like those of RNA viruses that lack enzymes allowing proofreading activity during replication (5). The main limitation of the proactive approach is cost and determining the extent of the investigations to undertake. Evaluating all insertions, deletions, and mutation combinations was cost-prohibitive in this study. A hybrid proactive/reactive model could be considered where all possible single-target mutations are assessed proactively, followed by a reactive approach for any mutation combinations or permutations that arise prospectively. However, even assessments of single mutations proactively would likely be cost-prohibitive in many laboratories. Alternative hybrid models could be envisioned, including a reactive approach paired with *in silico* analyses to help triage mutations anticipated to have a greater risk for NAAT target detection failure. Mutations at high risk for target detection failure include those near the 3’ end of primers that impede primer binding or extension by DNA polymerase, or viral genomes with two or more mutations, insertions, or deletions in a primer or probe binding sites (31 – 33). Regardless of the genetic surveillance strategy chosen, each approach should weigh the potential risks of diagnostic failure against the resources needed for implementation and sustainability.

Mutation impact characterization with custom synthetic DNAs was simple and circumvented previous challenges with clinical specimens such as sample depletion or difficulties in gaining access from laboratories worldwide. When compared to clinical specimens harboring the same NAAT target site mutations for the Xpert N2 gene target, synthetic DNAs showed near perfect concordance (*R* ^2^ = 0.9936; Table S3). For methods without a quantifiable output signal like the ID NOW assay (35), a quantifiable calibrator can be fused to the target of interest in the synthetic DNA to allow sample normalization and mutation impact assessment through serial dilution and endpoint comparisons. For real-time RT-PCR technologies like Xpert and Cobas (34), access to Ct values greatly facilitated sample normalization and mutation impact assessments as these acted as surrogates for virus concentration and rapidly identified mutations resulting in target detection failure or reduced sensitivity. For mutations resulting in sensitivity reduction, a greater than 3 SD cutoff from the expected Ct difference between the calibrator and target seemed like a reasonable starting point; however, depending on the NAAT application and risk of false negative results tolerated, higher cutoff values could be considered (1, 36).

When mutation impact assessment results were compared with those of published data or passive surveillance in Nova Scotia, some interesting observations were noted. First, the synthetic DNA approach reveals a single mutation [i.e., C26340T (9)] for the Cobas assay, but passive surveillance data and published reports demonstrated showed mutations with impacts either Orf1ab (i.e., G15006T [18] and T15009C [19]) or the E gene target (i.e., G26372T [20] and C26388T). These results suggest that for certain NAATs like the Cobas assay, clinical specimens or alternative methods (e.g., synthetic RNA) should be considered for mutation impact assessments. Regardless, the synthetic DNA approach was useful to characterize mutations impacting the N2 gene target of the Xpert assay.

**Fig 2:**
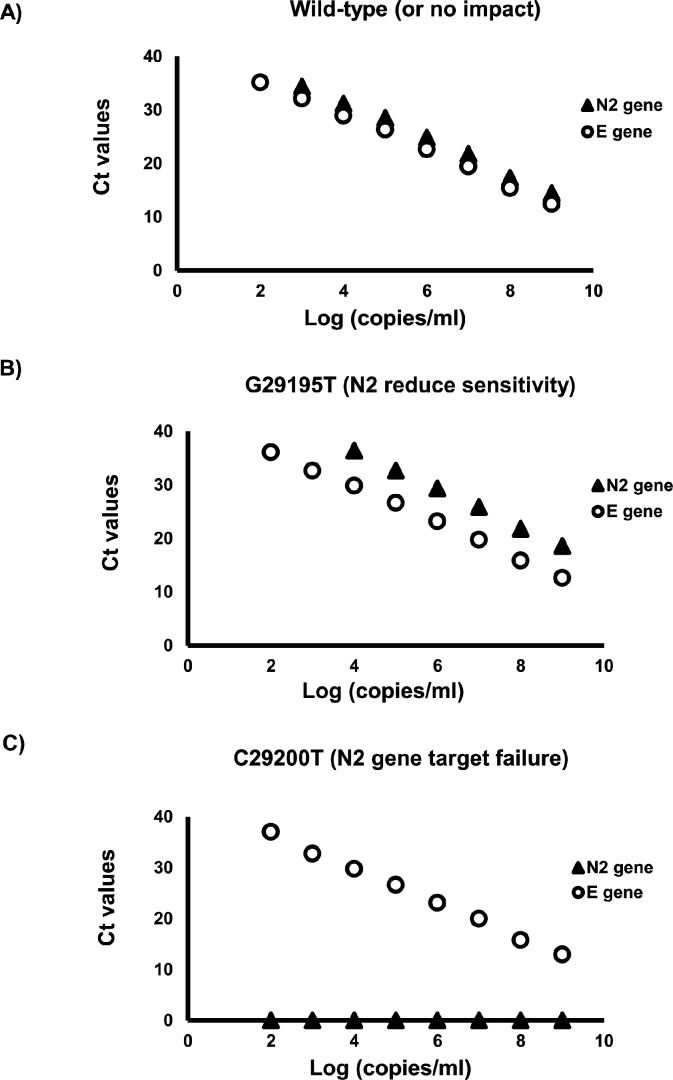
Dynamic range of the Xpert E and N2 gene targets. Ten-fold serial dilution of synthetic DNA harboring the wild-type E gene target sequence as a calibrator, and one of the following N2 target sequences: (A) wild-type; (B) a N2 G29195T mutant shown to reduce N2 target sensitivity; and (C) a C29200T mutation known to cause N2 target detection failure. Abbreviation: Ct, threshold cycle.

Other interesting observations were also noted. For example, no SARS-CoV-2 genomes in GISAID had a C29200A mutation despite it previously being reported to cause Xpert N2 gene target failure (13). This example highlights the lack of completeness of genomic databases, and submissions to international databases should be encouraged when a mutation compromising NAAT target detection is identified. Next, Ko et al. (21) observed a G29195T mutation that led to Xpert N2 gene target failure in SARS-CoV-2 positive specimens with high viral loads (i.e., E gene Ct values of 12.9 and 13.2). In this study, the G29195T mutation resulted in a consistent 14-fold reduction in sensitivity with normalized synthetic DNA concentrations (Table 3 and Table S4), and similar results were seen with three clinical specimens harboring this mutation (Table S3). In efforts to understand this discrepancy, 10-fold serial dilutions of synthetic DNA harboring the G29195T mutation were compared to a similar dilution series of wild-type DNA. For most dilutions, G29195T resulted in an approximately 10-fold reduction in sensitivity; however, sporadic N2 gene detection failures were observed near the detection limit and at high-mutated target concentrations (Fig. 2; Table S4), which is a well-recognized concept (34 – 37). However, inconsistencies at high viral concentrations are rarely described. Normalization of synthetic DNA concentration in the Xpert N2 gene target evaluation avoided misleading or erroneous results at extremely low- or high-target concentrations.

Assessments of the target site for the isothermal NAAT (i.e., Abbott ID NOW) were important, as any mutation could potentially lead to diagnostic failure in this single-target assay (35). Interestingly, none of the 23 mutations observed in its 36 bp target site compromised detection. While future studies should use clinical specimens to confirm this study’s findings from synthetic DNAs, it is possible that the low temperature of the ID NOW nicking enzyme amplification reaction (NEAR) technology might have helped tolerate some sequence mismatches (1). Even in the absence of mutations identified that compromise ID NOW RdRp detection, there is a need for ongoing genetic surveillance as other mutations alone or in combination could potentially compromise the assay. On a similar note, dual-target detection with the Xpert and Cobas real-time RT-PCRs reduces the potential of diagnostic failure but does not obviate the need for NAAT target site quality assurance (34, 35). LeBlanc et al. (37) demonstrated that not all NAAT targets are equivalent in terms of sensitivity for SARS-CoV-2 detection, and the loss of one target in a dual-target assay would render the assay susceptible to diagnostic failure. It could be argued that additional SARS-CoV-2 targets could be added to NAATs to mitigate the risk of NAAT false negative results (38, 39) but increasing NAAT sensitivity by incorporation of additional targets typically comes at the cost of an overall reduction in specificity, and vice versa. Regardless of possible mitigation strategies, ongoing surveillance for each target is important to ensure NAAT targets remain accurate over time. To emphasize the importance of monitoring over time, eight occurrences of the C26340T mutation impact the Cobas E gene target failure had been observed in the GISAID genome database at the time of this study, but 8,824 occurrences were observed with more recent analyses (on 2 April 2023). Ongoing NAAT target site mutation monitoring, mutation impact assessments, and communication with method users are important.

An anticipated argument against NAAT target site quality assurance is the perceived low prevalence of mutations impacting diagnostic detection. Comparing mutation occurrences against the millions of genome database entries is misleading and lacks mutated strain context and spatiotemporal distribution (9, 16, 39). For example, Lopez et al. (40) demonstrated high-local prevalence (8.5%) of the C29197T mutation causing Xpert N2 gene target failure, which was threefold higher than estimates for the entire state of California at the time. Recently, Isabel et al. (16) described the spatiotemporal distribution of the C29200T mutation in Canada, which appeared in 0.6% (266/41,712) of SARS-CoV-2 genomes. The true impact of this mutation was likely underestimated due to the small subset (i.e., 3.5%) of Canadian SARS-CoV-2 cases that had genome submitted to GISAID at the time. It is also interesting that the T15009C and C26388T mutations from the current study were in the alpha and omicron VOCs, highlighting the potential risk for strains with NAAT target site mutations to spread. Fortunately, public health quarantine measures limited the transmission of these mutated SARS-CoV-2 VOCs in this study. Altogether, targeted genetic surveillance should be encouraged when any NAAT target site mutation is identified, with consideration of potential local impacts and spatiotemporal distribution.

Another interesting but important topic for discussion is who should perform NAAT target site quality assurance. There are no mandatory requirements to undertake this endeavor or report deleterious mutations to manufacturers or regulatory authorities. A robust NAAT target site quality assurance program requires dedicated resources, and most laboratories would be challenged to provide ongoing up-to-date information for even one of the various commercially available NAATs. Given this burden, it should be questioned whether post-marketing target site quality assurance should be more rigorously enforced for each commercial NAAT. Some manufacturers do conduct NAAT target site quality assurance and products are reformulated if needed (39), but it is unclear to what extent these activities take place, as communication of these findings is rarely disclosed publicly. Ideally, a collaboration between clinical laboratories, regulatory authorities, and industry partners would ensure transparency and confidence in mutation impact assessments and target site quality assurance.

Other areas for future improvement would include the NGS pipeline to streamline the bioinformatics approach to identify mutations as early as SARS-CoV-2 sequence assembly and lineage determination. Communication could also be facilitated by the development of publicly accessible databases where mutations impacting NAATs could be cataloged, and international biorepositories of specimens harboring mutations (or cultured isolates of mutated strains) would greatly help assess the impact of NAAT target site mutations for diagnostic tests.

Overall, this study used SARS-CoV-2 as a model to demonstrate and discuss key considerations for a quality assurance framework for identifying, monitoring, and assessing the impact of target site mutations in commercial NAATs. Various challenges and solutions were presented to spark ongoing discussion on NAAT target site quality assurance, as these concepts could be applied to virtually any NAAT target, technology, or application. The question remains of who should undertake this responsibility to ensure the ongoing accuracy of commercial NAATs targets used for clinical diagnostics.

## MATERIALS AND METHODS

### Specimens and commercial NAATs

Clinical specimens were collected in universal transport media (Copan Diagnostics, Murietta, CA) and submitted for routine diagnostic testing at Nova Scotia Health (NSH; Halifax, NS) according to manufacturer recommendations on the Xpert Xpress SARS-CoV-2 assay on the GeneXpert instrument (Cepheid, Sunnyvale, CA), the Cobas 6800 SARS-CoV-2 assay on the Cobas 6800 instrument (Roche Diagnostics GmbH, Mannheim, Germany), or the Aptima SARS-CoV-2 Assay on the Panther System (Hologic Inc., San Diego, CA). The ID NOW COVID-19 assay (Abbott Diagnostics Inc., Scarborough, ME) was used for antigen result confirmation during community testing (35). Sequenced SARS-CoV-2 specimens with wild-type or mutations of interest were retrieved from −80°C archives at NSH or the National Microbiology Laboratory (NML; Winnipeg, MB). Synthetic DNAs were diluted in pooled test-negative specimens and processed as clinical specimens. Specimen pools were confirmed negative for SARS-CoV-2 by prior testing with each NAAT under investigation.

### NAAT-derived amplicon purification

Following Xpert testing with a positive SARS-CoV-2 specimen, a sterile disposable scalpel was used to excise the real-time RT-PCR chamber from the test device, and a 10 µL micropipette was used to retrieve the RT-PCR mixture containing the E and N2 gene target amplicons. A similar process was used to recover the amplicons derived from the amplification plate of the Cobas assay or the reaction cartridge of the ID NOW assay. Amplicons were subjected to 2% (wt/vol) agarose gel electrophoresis with 10 µg/mL ethidium bromide staining and were purified using a QIAquick Gel Extraction Kit QIAquick PCR and Gel Cleanup Kit (Qiagen GmbH, Hilden, Germany). Purified amplicons were subjected to NGS.

### NAAT target site identification using NGS

Purified amplicons from the Xpert and Cobas real-time RT-PCR assays were quantified using a Qubit 4 Fluorometer (ThermoFisher, Singapore) and normalized to a final concentration of 3.75 ng/µL. Library preparation was performed using a QIAseq 1-Step Amplicon Library Kit (Qiagen Inc., Hilden, Germany) using manufacturer guidelines, with the exception of the amplification stage, where five PCR cycles were chosen due to the large amount of starting material. Following library preparation, each library was quantified using a Qubit and diluted to 4 nM in 10 mM Tris-HCl (pH 8.0) with 0.1% Tween-20 and pooled in equal volumes. Following manufacturer guidelines, 5 µL of the pooled library was denatured with 5 µL of 0.2M NaOH and diluted to 7 pM with Illumina HT1 hybridization buffer in a final volume of 1 mL. Paired-end sequencing was conducted with a MiSeq Benchtop Sequencer using an MiSeq Nano Kit v2 (Illumina Inc., San Diego, CA).

Due to the isothermal NEAR (1) technology employed with the ID NOW assays, a modified adapter ligation approach was conducted. DNA end repair/dA tailing was performed using NEBNext Ultra II End Repair/dA-Tailing Module (New England Biolabs Ltd., Whitby, ON) using 1.2 µL of reaction buffer, 0.5 µL enzyme mix, 5 µL of PCR-grade water, and 3 µL of approximately 2 ng/µL gel purified target amplicon. This reaction was incubated at 20°C for 15 min. followed by a 65°C heat inactivation for 15 min. Adapters were provided from the Qiagen QIAseq 1-Step Amplicon Library Kit, and 1.25 µL of adapters, 5 µL of Blunt/TA ligase Master Mix (New England Biolabs), 3 µL of water, and 0.75 µL of end-repaired product from the previous step were mixed and incubated at 20°C for 20 min, followed by 65°C heat inactivation for 10 min. The resulting products were purified with AMPure XP beads (Beckman Coulter Inc., Brea, CA) using a ratio of 9:5 following standard manufacturer protocols, with final elution in 25 µL of water. PCR enrichment followed the same procedure as previously described for Cobas and Xpert targets, and bead-based was performed post-enrichment with a final elution in 30 µL of water. Illumina library preparation and sequencing steps were identical to those used for the real-time RT-PCR amplicons.

All library preparations were spiked 10% with Illumina PhiX Sequencing Control v3 and added to an Illumina MiSeq v2 Nano 300 cycle reagent cartridge for sequencing on an Illumina MiSeq benchtop sequencer. Sequence data were analyzed using the Signal pipeline (https://github.com/ayooluwaB/covid-19-signal-nml/blob/master/PIPELINE.md). NAAT target sites were annotated by alignment to the Wuhan-Hu-1 reference genome (Genbank accession number NC_045512.2) using a pairwise nucleotide Basic Local Alignment Search Tool function on the National Center for Biotechnology Information website (https://www.ncbi.nlm.nih.gov/).

### Query of SARS-CoV-2 genome databases for NAAT target site mutations

NAAT target sites were compared to SARS-CoV-2 genome sequences in GISAID (29) using PrimerChecker software version 3.03 (https://gisaid.org/). Given the exact primer and probes used in each NAAT remain proprietary, NAAT target sites deduced with NGS were split into three arbitrary segments to mimic the software input requirements for the primer (forward and reverse) and probe. Output data highlighted sequence mismatches between NAAT target sites and the corresponding SARS-CoV-2 sequences. Sequences with ambiguous bases were excluded.

### Mutation impact assessment

**Fig 3:**
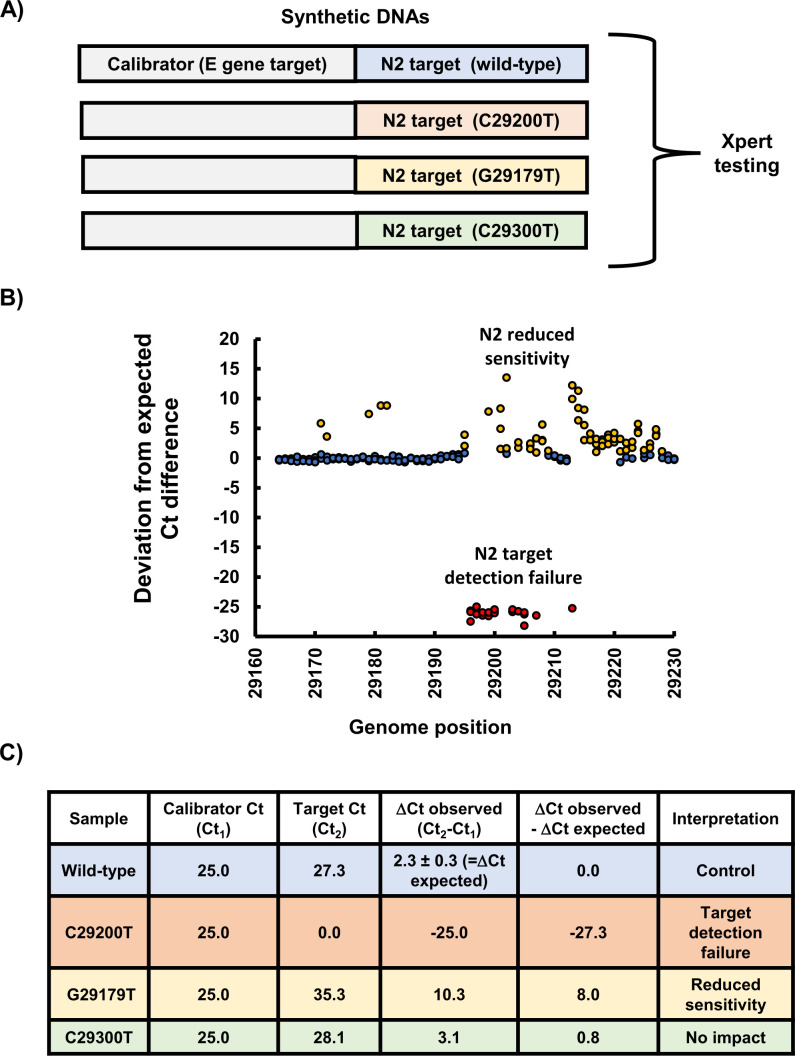
Dual target synthetic DNA-based mutation impact assessment. The principle of mutation impact assessment is illustrated using the Xpert N2 target site as an example. (**A**) Custom synthetic DNAs composed of a calibrator sequence that remains unchanged (e.g., Xpert E target) and either a wild-type or mutated target sequences (e.g., Xpert N2 target) are compared by NAAT testing following concentration normalization. The average Ct difference between the calibrator and wild-type target (i.e., expected ∆Ct) can be subtracted from those obtained with the calibrator and mutated targets (i.e., observed ∆Ct). (**B**) When plotted, target site mutations with no impact fall between ±3 SD of the average expected difference between wild-type calibrator and targets; those causing reduced sensitivity are above +3 SD; and mutations resulting in a target detection failure (target Ct = 0.0) result in negative values near −25, given synthetic DNAs are initially normalized to Ct values between 20 and 25. Abbreviations: cycle threshold (Ct) and nucleic acid amplification test (NAAT).

GeneArt Strings DNA Fragments (Thermo Fisher Scientific, Burlington, ON) were synthesized as concatenated fragments, fusing wild-type or mutated target site sequences to a calibrator sequence (used to normalize DNA concentration; Fig. 3A). Each synthetic DNA was confirmed by Sanger sequencing, normalized to 1 µM (to yield calibrator target Ct values between 20 and 25), diluted 1:10^6^ in pooled test-negative clinical specimens, and tested on the NAAT under investigation. For each NAAT target under investigation, a different calibrator sequence was used. To assess Xpert N2 gene target mutations, the Xpert E gene was used as a calibrator, and vice versa (Table 1). For the Cobas E gene, the Cobas RdRp target was used as a calibrator, and vice versa (Table 1). For the ID NOW RdRp target, the Cobas E gene was used as the calibrator (Table 1).

To characterize the impact of target site mutations for the Cobas and Xpert assays, threshold cycle (Ct) value comparisons were used. The expected Ct difference [Ct_2(WT)_ − Ct_1(CAL)_] between the calibrator [Ct_1(CAL)_] and wild-type (WT) target [Ct_2(WT)_] sequence was subtracted from those obtained with the mutated NAAT target sequences [Ct_2(mut)_] and its paired calibrator. Any deviation greater than 3 SD from the expected Ct differences of calibrator and WT sequences was considered reduced sensitivity, whereas lack of a Ct_2(MUT)_ value during testing was considered a target detection failure. Plotting data from the [Ct_2(MUT)_ − Ct_1(CAL)_] – [Ct_2(WT)_ − Ct_1(CAL)_] formula resulted in mutations leading to reduced sensitivity being visualized as positive signals outside the 3 SD cutoff, whereas target detection failures are seen as negative values in the −20 to −25 range (given the samples were normalized to Ct values between 20 and 25) (Fig. 3B). Fold reductions in sensitivity were calculated relative to a standard curve (Ct vs concentration) generated for each NAAT target with quantified gamma-irradiated SARS-CoV-2 provided by the NML (Winnipeg, MB, Canada) (41).

For NAATs without an output signal that is proportional to the viral concentration (e.g., Ct values), a permutation of this strategy can be employed. For example, to allow for quantification and normalization of the ID NOW-containing synthetic DNA, its target site sequence was fused to a calibrator (i.e., Cobas E gene) (Table 1), and dilutions of each fragment were tested on the Cobas assay. Following normalization from the resulting E gene Ct values, 10-fold serial dilutions were performed in test-negative specimen pools to compare detection endpoints on the ID NOW instrument.

### Genetic surveillance

Mutation monitoring can be passive, reactive, or proactive (Fig. 1). In passive surveillance, SARS-CoV-2 positive specimens identified from routine diagnostic testing displaying a greater than expected Ct shift between the targets of the Xpert or Cobas assays were selected for sequencing. In the reactive approach, the GISAID database was interrogated for SARS-CoV-2 variants with NAAT target mutations, and only sequences from these strains were subjected to subsequent impact assessments with synthetic DNAs. In the proactive strategy, all possible mutations that could occur in a NAAT target site are evaluated, followed by monitoring for the subset of mutations that are shown to compromise target detection. As a proof-of-principle, synthetic DNAs were designed that represented wild-type or 201 possible single mutations in the 67 bp Xpert N2 gene target site. Any mutation shown to compromise target detection was queried against local and national archives in attempts to confirm results using primary clinical specimens harboring the same mutation.

## Data Availability

Available data can be found in supplemental material.

## SUPPLEMENTAL MATERIAL

Table S1 spectrum.00761-23-s0001.pdf Passive surveillance mutations.

Table S2 spectrum.00761-23-s0002.pdf ID NOW mutation analysis.

Table S3 spectrum.00761-23-s0003.pdf Synthetic DNA versus clinical specimens.

Table S4 spectrum.00761-23-s0004.pdf High and low viral titer variation.

ASM does not own the copyrights to Supplemental Material that may be linked to, or accessed through, an article. The authors have granted ASM a non-exclusive, world-wide license to publish the Supplemental Material files. Please contact the corresponding author directly for reuse.
