# Supplementary material for: Neglected SARS-CoV-2 variants and potential concerns for molecular diagnostics: a framework for nucleic acid amplification test target site quality assurance
Source: Microbiol Spectr. 2023 Oct 10;11(6):e00761-23. doi: 10.1128/spectrum.00761-23 (PMC10715164; doi:10.1128/spectrum.00761-23)
Supplement: Table S1 — Passive surveillance mutations. [file spectrum.00761-23-s0001.pdf]

Table S1. Mutations impacting SARS-CoV-2 NAATs identified in Nova Scotia by passive surveillance.

NAAT	Target	Mutation	Impact	GISAID accession number	SARS-CoV-2 lineage
Cobas 6800 (Roche)	RdRp	T15009C	Reduced sensitivity	EPI_ISL_14746374	BA.2.12.1
	E	C26340T	Target detection failure	EPI_ISL_11623492	BA.1
		C26388T	Reduced sensitivity	EPI_ISL_5439011; EPI_ISL_5439104; EPI_ISL_10935261; EPI_ISL_10935262; EPI_ISL_10935265; EPI_ISL_10935266; EPI_ISL_10935267; EPI_ISL_10935268; EPI_ISL_10935269; EPI_ISL_10935276; EPI_ISL_10935296; EPI_ISL_10935314	B.1.1.7
Xpert (Cepheid)	N2	C29179T	Reduced sensitivity	EPI_ISL_915139	L.1
		C29197T	Target detection failure	EPI_ISL_1055493; EPI_ISL_1055498; EPI_ISL_10935669	B.1.1.519
		C29200T	Target detection failure	EPI_ISL_12686576; EPI_ISL_1055724	AY.122 AM.4

Abbreviation: envelope (E), nucleic acid amplification tests (NAAT), nucleocapsid (N), and RNA-dependent RNA polymerase (RdRp).
