# Supplementary material for: Neglected SARS-CoV-2 variants and potential concerns for molecular diagnostics: a framework for nucleic acid amplification test target site quality assurance
Source: Microbiol Spectr. 2023 Oct 10;11(6):e00761-23. doi: 10.1128/spectrum.00761-23 (PMC10715164; doi:10.1128/spectrum.00761-23)
Supplement: Table S2 — ID NOW mutation analysis. [file spectrum.00761-23-s0002.pdf]

Table S2. Mutation analysis for the ID NOW target site.

Mutation	Results for synthetic DNA dilutions					
	10^{-8}		10^{-9}		10^{-10}	
	Calibrator* Ct (avg. +/- SD)	Positive ID NOW results	Calibrator* Ct (avg. +/- SD)	Positive ID NOW results	Calibrator* Ct (avg. +/- SD)	Positive ID NOW results
**WT**	27.9 +/- 0.4	3/3	30.9 +/- 0.5	3/3	35.9 +/- 0.5	0/3
**T13370C**	28.0 +/- 0.3	3/3	31.1 +/- 0.1	3/3	36.1 +/- 0.4	0/3
**G13371T**	28.1 +/- 0.3	3/3	31.4 +/- 0.2	3/3	36.2 +/- 0.3	0/3
**G13371A**	28.1 +/- 0.3	3/3	31.3 +/- 0.2	3/3	36.2 +/- 0.2	1/3
**C13372T**	28.3 +/- 0.5	3/3	31.6 +/- 0.5	3/3	36.1 +/- 0.3	0/3
**A13373G**	28.4 +/- 0.4	3/3	31.6 +/- 0.4	3/3	36.4 +/- 0.4	0/3
**C13376T**	27.8 +/- 0.4	3/3	31.3 +/- 0.1	3/3	36.3 +/- 0.1	0/3
**T13382C**	28.2 +/- 0.3	3/3	31.1 +/- 0.1	3/3	36.1 +/- 0.3	0/3
**T13384G**	28.0 +/- 0.2	3/3	31.0 +/- 0.3	3/3	36.2 +/- 0.2	0/3
**T13384C**	27.9 +/- 0.3	3/3	31.6 +/- 0.4	3/3	36.3 +/- 0.5	0/3
**T13385G**	28.1 +/- 0.4	3/3	31.0 +/- 0.3	3/3	36.1 +/- 0.4	0/3
**T13386G**	28.1 +/- 0.1	3/3	31.1 +/- 0.2	3/3	36.1 +/- 0.2	0/3
**A13388G**	28.2 +/- 0.2	3/3	31.3 +/- 0.4	3/3	36.3 +/- 0.3	0/3
**T13389C**	28.1 +/- 0.2	3/3	31.2 +/- 0.2	3/3	36.2 +/- 0.3	0/3
**C13391T**	28.0 +/- 0.2	3/3	30.9 +/- 0.4	3/3	36.0 +/- 0.4	0/3
**T13394C**	27.8 +/- 0.4	3/3	31.4 +/- 0.2	3/3	36.1 +/- 0.3	0/3
**A13395G**	27.9 +/- 0.3	3/3	30.8 +/- 0.3	3/3	35.9 +/- 0.5	0/3
**C13396T**	28.3 +/- 0.3	3/3	31.5 +/- 0.5	3/3	36.2 +/- 0.3	0/3
**A13397G**	28.0 +/- 0.2	3/3	30.9 +/- 0.4	3/3	35.9 +/- 0.6	0/3
**G13398T**	28.2 +/- 0.3	3/3	31.8 +/- 0.4	3/3	36.6 +/- 0.6	0/3
**T13399C**	27.8 +/- 0.5	3/3	31.2 +/- 0.1	3/3	36.2 +/- 0.4	0/3
**G14400T**	28.1 +/- 0.4	3/3	31.7 +/- 0.5	3/3	36.4 +/- 0.5	0/3
**G14400A**	27.9 +/- 0.3	3/3	31.2 +/- 0.2	3/3	36.1 +/- 0.3	0/3
**C14403T**	28.1 +/- 0.2	3/3	31.5 +/- 0.4	3/3	36.2 +/- 0.4	0/3

*The calibrator for the ID NOW target mutation assessment was the Cobas E gene. Each Synthetic DNA was run on the Cobas 6800 instrument and synthetic DNA concentrations were normalized with Ct values ~28, which corresponds to approximately 1000-fold more than the assay limit of detection.
