# Supplementary material for: Neglected SARS-CoV-2 variants and potential concerns for molecular diagnostics: a framework for nucleic acid amplification test target site quality assurance
Source: Microbiol Spectr. 2023 Oct 10;11(6):e00761-23. doi: 10.1128/spectrum.00761-23 (PMC10715164; doi:10.1128/spectrum.00761-23)
Supplement: Table S3 — Synthetic DNA versus clinical specimens. [file spectrum.00761-23-s0003.pdf]

Table S3. Correlation between synthetic DNAs and SARS-CoV-2 positive clinical specimens harboring Xpert N2 mutations.

Lineage	Mutation	GISAID accession number	Clinical specimens					Synthetic DNAs					Concordant
			Ct (E)	Ct (N2)	ΔCt (N2-E)	Fold sensitivity reduction	Result	Ct (E)	Ct (N2)	ΔCt (N2-E)	Fold sensitivity reduction	Result	
AY.25.1	T29164C	EPI_ISL_12686696	30.2	33.3	3.1	1.8	No impact	21.6	23.5	1.9	0.8	No impact	Y
AY.25.1	T29164C	EPI_ISL_12686882	33.2	35.9	2.7	1.4	No impact	21.6	23.5	1.9	0.8	No impact	Y
BE.1.1	C29167T	EPI_ISL_11755769	25.8	27.7	1.9	0.8	No impact	21.8	23.8	2.0	0.9	No impact	Y
BA.2.12.1	C29167T	EPI_ISL_14152623	15.7	17.9	2.2	1.0	No impact	21.8	23.8	2.0	0.9	No impact	Y
BA.5.2	C29171T	N/A	25.6	33.2	7.6	38.5	Reduced sensitivity	23.2	31.2	8.0	50.5	Reduced sensitivity	Y
BA.5.2.1	C29171T	EPI_ISL_14734643	18.9	27.2	8.3	61.9	Reduced sensitivity	23.2	31.2	8.0	50.5	Reduced sensitivity	Y
AY.122	A29172G	N/A	28.4	34.2	5.8	11.4	Reduced sensitivity	22.2	27.8	5.6	10.7	Reduced sensitivity	Y
BA.1.1	A29172G	EPI_ISL_12687555	22.1	27.7	5.6	10.0	Reduced sensitivity	22.2	27.8	5.6	10.7	Reduced sensitivity	Y
BA.2	T29173C	EPI_ISL_12906704	19.4	21.6	2.2	1.0	No impact	21.5	23.8	2.3	1.1	No impact	Y
AY.73	G29179A	EPI_ISL_13287862	20.5	25.5	5.0	6.6	Reduced sensitivity	21.8	26.5	4.7	5.4	Reduced sensitivity	Y
AY.73	G29179A	EPI_ISL_12392769	27.8	32.5	4.7	5.4	Reduced sensitivity	21.8	26.5	4.7	5.4	Reduced sensitivity	Y
L.1	G29179T	EPI_ISL_12387584	25.6	35.2	9.6	149.0	Reduced sensitivity	22.1	31.7	9.6	149.0	Reduced sensitivity	Y
B.1.427	G29179T	EPI_ISL_13287232	18.3	28.2	9.9	182.5	Reduced sensitivity	22.1	31.7	9.6	149.0	Reduced sensitivity	Y
AY.27	G29179T	EPI_ISL_12389261	18.1	27.7	9.6	149.0	Reduced sensitivity	22.1	31.7	9.6	149.0	Reduced sensitivity	Y
AY.46	G29179T	N/A	25.6	34.8	9.2	113.7	Reduced sensitivity	22.1	31.7	9.6	149.0	Reduced sensitivity	Y
BA.2	A29181T	EPI_ISL_13287350	24.9	35.9	11.0	384.1	Reduced sensitivity	21.6	32.6	11	384.1	Reduced sensitivity	Y
B.1.1.7	A29182G	EPI_ISL_12387394	21.8	32.8	11.0	384.1	Reduced sensitivity	22.4	33.4	11	384.1	Reduced sensitivity	Y
B.1.438.1	G29195T	EPI_ISL_12387603	18.1	24.1	6.0	13.1	Reduced sensitivity	22.1	28.2	6.1	14.0	Reduced sensitivity	Y
AY.122	G29195T	EPI_ISL_12686575	17.2	23.4	6.2	15.0	Reduced sensitivity	22.1	28.2	6.1	14.0	Reduced sensitivity	Y
AY.122	G29195T	EPI_ISL_12686576	29.5	35.6	6.1	14.0	Reduced sensitivity	22.1	28.2	6.1	14.0	Reduced sensitivity	Y
UKN	C29197A	N/A	30.4	0.0	-30.4	n/a	Target failure	21.9	0.0	-21.9	n/a	Target failure	Y
B.1.1.519	C29197T	EPI_ISL_10935669	24.5	0.0	-24.5	n/a	Target failure	21.4	0.0	-21.4	n/a	Target failure	Y
AY.103	C29197T	EPI_ISL_12686700	26.8	0.0	-26.8	n/a	Target failure	21.4	0.0	-21.4	n/a	Target failure	Y
BA.5.2.1	C29200A	N/A	20.6	0.0	-20.6	n/a	Target failure	23	0.0	-23	n/a	Target failure	Y
BF.10	C29200T	EPI_ISL_14980816	19.4	0.0	-19.4	n/a	Target failure	21.7	0.0	-21.7	n/a	Target failure	Y
BA.1.1	G29202A	EPI_ISL_13287961	27.7	32	4.3	4.1	Reduced sensitivity	21.6	26.3	4.7	5.4	Reduced sensitivity	Y
B.1.1.7	C29203T	EPI_ISL_12387340	20.3	0.0	-20.3	n/a	Target failure	21.4	0.0	-21.4	n/a	Target failure	Y
B.1.1.7	C29203T	EPI_ISL_12387475	24.5	0.0	-24.5	n/a	Target failure	21.4	0.0	-21.4	n/a	Target failure	Y

BA.1.1	G29210T	EPI_ISL_12393487	22.9	25.5	2.6	1.3	No impact	21.3	23.6	2.3	0.0	No impact	Y
AY.106	G29212A	EPI_ISL_12388811	21.4	23.8	2.4	1.1	No impact	21.1	22.8	1.7	0.7	No impact	Y
BA.2	G29212A	EPI_ISL_12394209	18.2	20.6	2.4	1.1	No impact	21.1	22.8	1.7	0.7	No impact	Y
BA.5.2.1	G29212T	N/A	21.4	23.1	1.7	0.7	No impact	22.2	24.3	2.1	0.9	No impact	Y
BA.1.1	T29223C	EPI_ISL_12391001	33.4	39.3	5.9	12.2	Reduced sensitivity	23	29	6.0	13.1	Reduced sensitivity	Y
AY.78	G29227A	EPI_ISL_12686660	25.7	31.5	5.8	11.4	Reduced sensitivity	21.7	26.5	4.8	5.8	Reduced sensitivity	Y
BA.1.1	G29227A	EPI_ISL_12394682	24.8	30.7	5.9	12.2	Reduced sensitivity	21.7	26.5	4.8	5.8	Reduced sensitivity	Y
B.1.1.7	G29227T	EPI_ISL_12388686	19.1	24.5	5.4	8.7	Reduced sensitivity	21.9	27.3	5.4	8.7	Reduced sensitivity	Y
AY.126	G29227T	N/A	19.7	25.1	5.4	8.7	Reduced sensitivity	21.9	27.3	5.4	8.7	Reduced sensitivity	Y
BA.2.12.1	G29229A	EPI_ISL_13703622	21.9	23.3	1.4	0.6	No impact	21.5	23.9	2.4	1.0	No impact	Y
BA.1	C29230T	N/A	25.3	27.3	2.0	0.9	No impact	21.6	23.6	2.2	1.0	No impact	Y
BA.1	C29230T	EPI_ISL_9822104	24.1	25.7	1.6	0.7	No impact	21.6	23.6	2.2	1.0	No impact	Y
