# Supplementary material for: Neglected SARS-CoV-2 variants and potential concerns for molecular diagnostics: a framework for nucleic acid amplification test target site quality assurance
Source: Microbiol Spectr. 2023 Oct 10;11(6):e00761-23. doi: 10.1128/spectrum.00761-23 (PMC10715164; doi:10.1128/spectrum.00761-23)
Supplement: Table S4 — High and low viral titer variation. [file spectrum.00761-23-s0004.pdf]

Table S4. Dynamic range of Xpert with wild-type or the G29295T and C29200T mutants.

Strain	Dilutions	E gene Ct values		N2 gene Ct values		Δ Ct (N2-E)	Overall result	Comments
		Replicates (1/2/3)	Avg. ± SD	Replicates (1/2/3)	Avg. ± SD	Avg. ± SD		
Wild-type	10^{-1}	9.9/9.6/9.6	9.8 ± 0.2	11.0/11.6/11.2	11.3 ± 0.3	1.6 ± 0.5	Invalid (3/3)	Invalid
	10^{-2}	10.2/10.1/10.3	10.2 ± 0.1	11.6/11.8/11.5	11.7 ± 0.1	1.5 ± 0.2	Positive (3/3)	
	10^{-3}	12.4/12.5/12.7	12.5 ± 0.2	14.5/14.4/14.7	14.5 ± 0.2	2.0 ± 0.1	Positive (3/3)	
	10^{-4}	15.4/15.4/15.3	15.4 ± 0.1	17.3/17.4/17.4	17.4 ± 0.1	2.0 ± 0.1	Positive (3/3)	
	10^{-5}	19.5/19.4/19.5	19.5 ± 0.1	21.7/21.8/21.7	21.8 ± 0.1	2.3 ± 0.1	Positive (3/3)	
	10^{-6}	22.6/22.7/22.6	22.7 ± 0.1	24.8/24.9/24.8	24.9 ± 0.1	2.2 ± 0.0	Positive (3/3)	
	10^{-7}	26.3/26.4/26.3	26.4 ± 0.1	28.4/28.6/28.4	28.5 ± 0.1	2.1 ± 0.1	Positive (3/3)	
	10^{-8}	28.9/29.0/28.9	29.0 ± 0.1	31.1/31.3/31.1	31.2 ± 0.1	2.2 ± 0.1	Positive (3/3)	
	10^{-9}	31.9/32.4/32.7	32.2 ± 0.4	34.3/34.5/35.1	34.4 ± 0.4	2.3 ± 0.2	Positive (3/3)	N2 detection limit
	10^{-10}	34.3/36.0/37.1	35.2 ± 1.4	36.7/0.0/0.0	18.4 ± 21.2	2.2 ± 0.2	Presumptive positive (2/3)	E gene detection limit
	10^{-11}	39.7/42.0/40.0	41.1 ± 23.7	0.0/0.0/0.0	0.0 ± 0.0	-39.9 ± 1.3	Presumptive positive (2/3)	Inconsistent E
	10^{-12}	0.0/0.0/0.0	0.0 ± 0.0	0.0/0.0/0.0	0.0 ± 0.0	0.0 ± 0.0	Negative (3/3)	
G29195 T	10^{-1}	9.3/9.2/9.3	9.3 ± 0.1	11.6/12.2/0.0	11.9 ± 0.1	-1.3 ± 6.9	Invalid (2/3)	Inconsistent N2
	10^{-2}	10.1/10.2/10.2	10.2 ± 0.1	0.0/13.9/13.9	7.0 ± 0.1	-0.9 ± 8.0	Positive (2/3)	Inconsistent N2
	10^{-3}	12.8/12.4/12.6	12.6 ± 0.2	18.7/18.5/18.6	18.6 ± 0.2	6.0 ± 0.1	Positive (3/3)	
	10^{-4}	15.9/15.8/15.9	15.9 ± 0.1	21.8/21.8/21.9	21.8 ± 0.1	6.0 ± 0.1	Positive (3/3)	
	10^{-5}	19.8/19.7/20.0	19.8 ± 0.2	25.9/25.8/26.1	25.9 ± 0.2	6.1 ± 0.0	Positive (3/3)	
	10^{-6}	23.1/23.3/23.4	23.2 ± 0.2	29.2/29.5/29.5	29.4 ± 0.2	6.1 ± 0.1	Positive (3/3)	
	10^{-7}	26.5/26.8/26.8	26.7 ± 0.2	32.6/32.8/32.9	32.7 ± 0.2	6.1 ± 0.1	Positive (3/3)	
	10^{-8}	29.7/30.0/30.1	29.9 ± 0.2	36.4/36.4/36.4	36.4 ± 0.0	6.5 ± 0.2	Positive (3/3)	N2 detection limit
	10^{-9}	32.9/32.4/33.2	32.7 ± 0.4	38.7/0.0/0.0	19.4 ± 0.4	7.8 ± 0.8	Positive (2/3)	Inconsistent N2

	10^{-10}	35.6/36.6/36.6	36.1 ± 0.6	0.0/0.0/0.0	0.0 ± 0.6	-36.3 ± 0.6	Presumptive positive (3/3)	E gene detection limit
	10^{-11}	0.0/0.0/0.0	0.0 ± 23.0	0.0/0.0/0.0	0.0 ± 23.0	-13.4 ± 23.3	Negative (2/3)	Inconsistent E
	10^{-12}	0.0/0.0/0.0	0.0 ± 0.0	0.0/0.0/0.0	0.0 ± 0.0	0.0 ± 0.0	Negative (3/3)	
C29200T	10^{-1}	9.2/9.3/9.6	9.3 ± 0.2	0.0/0.0/0.0	0.0 ± 0.0	-9.4 ± 0.2	Invalid (2/3)	Invalid
	10^{-2}	10.7/10.3/10.5	10.5 ± 0.2	0.0/0.0/0.0	0.0 ± 0.0	-10.5 ± 0.2	Presumptive positive (3/3)	
	10^{-3}	12.9/13.0/13.2	13.0 ± 0.2	0.0/0.0/0.0	0.0 ± 0.0	-13.0 ± 0.2	Presumptive positive (3/3)	
	10^{-4}	15.7/15.9/16.0	15.8 ± 0.2	0.0/0.0/0.0	0.0 ± 0.0	-15.9 ± 0.2	Presumptive positive (3/3)	
	10^{-5}	19.9/20.0/20.3	20.0 ± 0.2	0.0/0.0/0.0	0.0 ± 0.0	-20.1 ± 0.2	Presumptive positive (3/3)	
	10^{-6}	23.0/23.2/23.4	23.1 ± 0.2	0.0/0.0/0.0	0.0 ± 0.0	-23.2 ± 0.2	Presumptive positive (3/3)	
	10^{-7}	26.5/26.7/26.8	26.6 ± 0.2	0.0/0.0/0.0	0.0 ± 0.0	-26.7 ± 0.2	Presumptive positive (3/3)	
	10^{-8}	29.4/30.1/30.0	29.8 ± 0.4	0.0/0.0/0.0	0.0 ± 0.0	-29.8 ± 0.4	Presumptive positive (3/3)	
	10^{-9}	32.4/33.1/32.6	32.8 ± 0.4	0.0/0.0/0.0	0.0 ± 0.0	-32.7 ± 0.4	Presumptive positive (3/3)	
	10^{-10}	36.8/37.3/38.2	37.1 ± 0.7	0.0/0.0/0.0	0.0 ± 0.0	-37.4 ± 0.7	Presumptive positive (3/3)	
	10^{-11}	43.1/0.0/0.0	21.6 ± 24.4	0.0/0.0/0.0	0.0 ± 0.0	-28.1 ± 24.4	Negative (2/3)	Inconsistent E
	10^{-12}	0.0/0.0/0.0	0.0 ± 0.0	0.0/0.0/0.0	0.0 ± 0.0	0.0 ± 0.0	Negative (3/3)	
